# DNA polymerases as useful reagents for biotechnology – the history of developmental research in the field

**DOI:** 10.3389/fmicb.2014.00465

**Published:** 2014-08-29

**Authors:** Sonoko Ishino, Yoshizumi Ishino

**Affiliations:** Department of Bioscience and Biotechnology, Graduate School of Bioresource and Bioenvironmental Sciences, Kyushu UniversityFukuoka, Japan

**Keywords:** thermostability, gene amplification, *in vitro* gene manipulation, Archaea, hyperthermophile

## Abstract

DNA polymerase is a ubiquitous enzyme that synthesizes complementary DNA strands according to the template DNA in living cells. Multiple enzymes have been identified from each organism, and the shared functions of these enzymes have been investigated. In addition to their fundamental role in maintaining genome integrity during replication and repair, DNA polymerases are widely used for DNA manipulation *in vitro*, including DNA cloning, sequencing, labeling, mutagenesis, and other purposes. The fundamental ability of DNA polymerases to synthesize a deoxyribonucleotide chain is conserved. However, the more specific properties, including processivity, fidelity (synthesis accuracy), and substrate nucleotide selectivity, differ among the enzymes. The distinctive properties of each DNA polymerase may lead to the potential development of unique reagents, and therefore searching for novel DNA polymerase has been one of the major focuses in this research field. In addition, protein engineering techniques to create mutant or artificial DNA polymerases have been successfully developing powerful DNA polymerases, suitable for specific purposes among the many kinds of DNA manipulations. Thermostable DNA polymerases are especially important for PCR-related techniques in molecular biology. In this review, we summarize the history of the research on developing thermostable DNA polymerases as reagents for genetic manipulation and discuss the future of this research field.

## IN THE BEGINNING: TAQ POLYMERASE

DNA polymerase I from *Thermus aquaticus* (Taq polymerase) is the most famous representative enzyme among the thermostable DNA polymerases. Taq polymerase was identified from* T. aquaticus* isolated from Yellowstone National Park in Montana, USA. The report was published by Chien et al. (1976) as her Master’s course study. At that time, nobody foresaw how famous this enzyme would later become. In 1985, PCR (polymerase chain reaction) technology using the Klenow fragment of DNA polymerase I from *Escherichia coli* was reported (Saiki et al., 1985). It was easily imagined that a heat-stable DNA polymerase that is not inactivated at the denaturation step from double-stranded to single-stranded DNA would transform this method of gene amplification to a practical technology. Subsequently, a simple and robust PCR method using Taq polymerase was published (Saiki et al., 1988). Due to the heat stability of Taq polymerase, the reaction tube could remain in the incubator after the reaction mixture containing the DNA polymerase was prepared, and only temperature changes were required for PCR. An instrument capable of quick reaction temperature change was developed, and the PCR market opened with a PCR kit (GeneAmp PCR Reagent Kit) and an instrument (Thermal Cycler) provided by Perkin-Elmer Cetus. DNA polymerase from *Thermus thermophilus* (Tth polymerase) was also developed as a commercial product in the early age of the PCR, but a scientific report was only an abstract of ASBMB in 1974 from the Mitsubishi-Kasei Institute of Life Sciences, Japan, where this enzyme was originally identified. A specific property of Tth polymerase is that it has a distinct reverse transcriptase (RT) activity, and a single-tube RT-PCR method was developed with this enzyme.

At the beginning of the PCR age, Taq polymerase was purified from *T. aquaticus* cells. However, the *pol* gene was soon cloned from the *T. aquaticus* genome and expressed in *E. coli* cells. The native Taq polymerase was replaced by the recombinant Taq polymerase, named AmpliTaq DNA polymerase, in the commercial field. The amount of the recombinant Taq polymerase produced in *E. coli* cells was very low, probably because of the low expression of the *T. aquaticus* gene, which has a high GC content (70%)*,* although the protein quality was improved, as compared to the native Taq polymerase (Lawyer et al., 1989). We successfully constructed an efficient overproduction system by changing the codons around the N-terminal region from the original gene to either the AT-type at the third letter or the optimal codons for *E. coli*. These manipulations improved the production of Taq polymerase more than 10-fold, as compared with the production of AmpliTaq (Ishino et al., 1994). Taq polymerase has been used as the standard enzyme for PCR since its inception. An abundance of PCR data obtained using Taq polymerase has been accumulated, providing a valuable resource for developing new products for useful PCR modifications.

## THERMOSTABLE DNA POLYMERASES FROM THERMOPHILES

**FIGURE 1:**
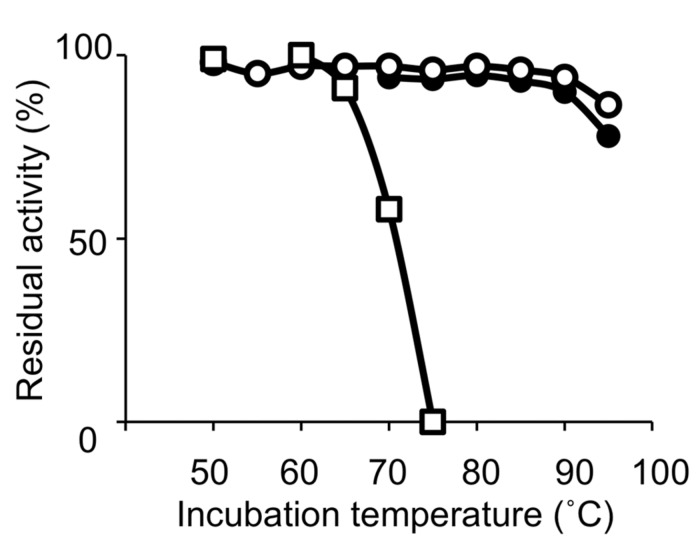
**Heat resistance of the DNA polymerases.** Residual DNA polymerase activities after incubation at the indicated temperature for 30 min were plotted. DNA polymerases from *Pyrococcus furiosus* (open circles), *Thermus aquaticus* (closed circles), and *Bacillus caldotenax* (open squares) were used as representatives from hyperthermophiles, extreme thermophiles, and moderate extremophiles, respectively.

Thermophilic organisms utilize thermostable DNA polymerases, and therefore, thermophiles became more popular as genetic resources of DNA polymerases and other enzymes for industrial use. The heat stability of the enzymes is directly related to the temperature, at which the organism thrives. Thermophiles are classified into extreme thermophiles, which grow at temperatures greater than 75^∘^C, and moderate thermophiles, which grow at 55–75^∘^C. The thermostabilities are obviously different between the DNA polymerases from extreme thermophiles and moderate thermophiles as shown in **Figure 1**. Taq polymerase is applicable to PCR; however, the DNA polymerases from the moderately thermophilic *Bacillus* species are not suitable for PCR, because of their insufficient stability. Hyperthermophiles are particular extreme thermophiles that grow optimally at temperatures above 80^∘^C. Most of the hyperthermophilic organisms are Archaea, although some are bacteria, as shown in (**Table 1**). Generally, hyperthermophiles have the potential to provide more heat-stable enzymes than normal thermophiles. Actually, the DNA polymerase from *Pyrococcus furiosus* (Pfu polymerase) is more stable than Taq polymerase (**Figure 1**). Hyperthermophilic archaea became popular not only as sources of useful enzymes for application, but also as interesting model organisms for molecular biology. In the early 1990s, the metabolic phenomena in archaeal cells were just barely understood, and therefore, the molecular biology of Archaea, the third domain of life, became a novel and exciting field.

**Table 1:** Representative hyperthermophiles.

	Growth conditions		
	Temperature		
Species	Min. temp to Max. temp (°C)	Opt. temp (°C)	Aerobic(ae)/Anaerobic(an)
**Archaea**
**Crenarchaeota**			
*Acidianus infernus*	60–95	88	ae/an
*Sulfolobus acidocaldarius*	60–85	75	ae
*Pyrobaculum islandicum*	74–103	100	an
*Thermoproteus tenax*	70–97	88	an
*Desulfurococcus mobilis*	70–95	85	an
*Aeropyrum pernix*	70–100	90	ae
*Ignicoccus islandicus*	65–100	90	an
*Pyrolobus fumarii*	90–113	106	ae/an
**Euryarchaeota**			
*Pyrococcus furiosus*	70–105	100	an
*Thermococcus kodakarensis*	60–100	85	an
*Archaeoglobus fulgidus*	60–95	83	an
*Methanopyrus kandleri *	90–122	105	an
*Methanothermus sociabilis*	65–97	88	an
*Methanococcus igneus*	45–91	88	an
**Bacteria**
*Thermotoga maritima*	55–90	80	an
*Aquifex pyrophilus*	67–95	85	ae

## DNA POLYMERASES FROM HYPERTHERMOPHILES

When choosing thermostable DNA polymerases as reagents for genetic engineering, research scientists generally do not consider the biology of the source organisms. The properties of the obtained enzyme are important, regardless of the source. To obtain a thermostable DNA polymerase, the growth temperature of the thermophile attracts the most attention. *Thermotoga maritima* DNA polymerase was the first commercial product (ULTIMA DNA polymerase) from the hyperthermophilic bacteria. This enzyme has an associated 3^′^–5^′^ exonuclease activity and thus is expected to perform PCR more accurately with its proofreading activity. All PCR enzymes from the domain Bacteria are from family A, whose members generally lack 3^′^–5^′^ exonuclease activity, and ULTMA DNA polymerase was an exception, like *E. coli* Pol I. In spite of this selling point, ULTIMA DNA polymerase was not a commercial success. One report described no significant differences in the fidelities of the ULTIMA and Taq polymerases, when using optimal buffer conditions for each enzyme, for sequencing purposes (Diaz and Sabino, 1998).

DNA polymerases from the hyperthermophilic archaea were also assessed as PCR enzymes. We cloned the *pol* gene from *P. furiosus* and expressed it in *E. coli* (Uemori et al., 1993). We thought ours would be the first report of the full-length sequence of an archaeal family B DNA polymerase, which had been predicted earlier because of the aphidicolin-sensitive phenotype of a halophile and a methanogen (Forterre et al., 1984; Zabel et al., 1985). However, two papers showing the deduced total amino acid sequences of DNA polymerases from the hyperthermophilic archaea, *Sulfolobus solfataricus* (Pisani et al., 1992) and *Thermococcus litoralis* (Perler et al., 1992) were published during the preparation of our manuscript (Uemori et al., 1993). All these reports clearly showed that the archaeal DNA polymerases have sequences similar to the eukaryotic replicative DNA polymerases, Pol α, δ, and ε (family B). It is also interesting that the *T. litoralis pol* has inteins that must be spliced out after translation (Perler et al., 1992). Thereafter, many cases of DNA polymerases containing various pattern of inteins, inserted in motifs A, B, and C, were discovered (Perler, 2002). The fidelity of DNA synthesis *in vitro* is markedly affected by the reaction condition. However, the archaeal family B enzymes generally perform more accurate DNA synthesis as compared with Taq polymerase (Cariello et al., 1991; Ling et al., 1991; Lundberg et al., 1991; Mattila et al., 1991), suggesting that the strong 3^′^–5^′^ exonuclease activities of the hyperthermophilic family B polymerase *in vitro* affect the fidelity of PCR.

## DEVELOPMENT OF LA-PCR

**FIGURE 2:**
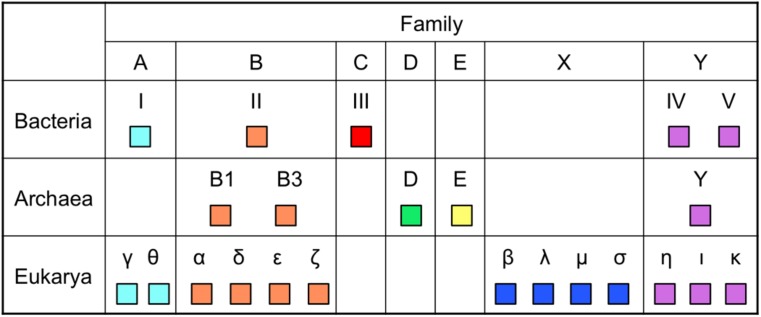
**Distribution of DNA polymerases in the three domains of life.** The names of DNA polymerases vary, depending on the domains. Only DNA polymerases with *in vitro* activity, if applicable, are shown. Eukaryotic Polγ is from mitochondria and archaeal PolE is a plasmid-encoded enzyme.

DNA polymerases are classified into seven families based on the amino acid sequence similarity (**Figure 2**). To date, the enzymes utilized for genetic engineering have been only from families A and B among them. Taq polymerase from family A has strong extension ability and performs efficient amplification of the target DNA. However, their fidelity is low. On the other hand, the Pfu polymerase from family B performs highly accurate PCR amplification, but their extension rate is slow and a long extension time is required for each cycle of PCR. Therefore, a method was required for the accurate PCR amplification of long DNA regions. One simple idea that researchers considered trying was to combine one enzyme each from family A and family B in a single PCR reaction mixture. However, the actual PCR performance was not so simple, and persevering trials were necessary to find suitable conditions to develop a long and accurate (LA) PCR system. The amplification of a ∼35 kb DNA fragment from λ phage genomic DNA was successfully accomplished in 1994, by the mixture of Klentaq1 (N-terminal deletion mutant of Taq polymerase) and an archaeal family B DNA polymerase with 3^′^–5^′^ exonuclease activity (Barns, 1994). Subsequently, commercial products for LA-PCR were rapidly developed by several manufactures and LA-PCR technology became popular throughout the world.

## FAST AND HIGHLY ACCURATE PCR BY AN ARCHAEAL FAMILY B DNA POLYMERASE

A family B DNA polymerase from the hyperthermophilic archaeon, *Thermococcus kodakarensis* (this strain was originally named *Pyrococcus kodakaraensis* KOD1), was identified and applied to PCR (Takagi et al., 1997). This enzyme has the typical amino acid sequence of the archaeal family B enzymes, but it showed a high extension rate while maintaining high fidelity, and therefore, the commercial product, KOD DNA polymerase (KOD Pol), was developed and became popular as a PCR enzyme. Commercial products related to KOD Pol, including a hot start kit with a monoclonal antibody and an LA-PCR kit with a mixture of the wild type and 3^′^–5^′^ exonuclease-deficient mutant of this enzyme, were subsequently developed by the manufacturers. The underlying reason why this family B enzyme shows high extension speed is interesting. Comparisons of the crystallographic structures and amino acid sequences of KOD Pol with other archaeal family B enzymes revealed the logical explanation for the efficient extension ability of this enzyme. Many basic residues are located around the active site in the finger domain of KOD Pol. In addition, many Arg residues are located at the forked point, which is the predicted as the junction of the template binding region and the editing cleft. This unique structure may stabilize the melted DNA structure at the forked point, resulting in high PCR performance (Hashimoto et al., 2001).

## BASIC RESEARCH ON ARCHAEAL DNA POLYMERASES

**FIGURE 3:**
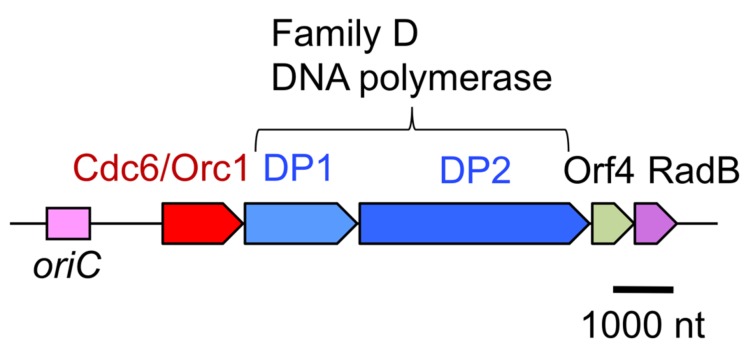
**Physical map of the *P. furiosus* chromosomal segment bearing the replication origin and the replication proteins.** The genes encoding PolD, the archaea-specific DNA polymerase, is located near the replication origin, *oriC*, and are transcribed as an operon with the initiator protein, Cdc6/Orc1, and a recombination protein, RadB.

Research on DNA polymerases in hyperthermophilic archaea is motivated by not only industrial applications, but also basic molecular biology, to elucidate the molecular mechanisms of genetic information processing systems at extremely hot temperatures. To identify all of the DNA polymerases in the archaeal cell, we tried to separate the DNA polymerase activities in the total cell extract of *P. furiosus*. Three major fractions showed nucleotide incorporation activity after anion exchange column chromatography (Resource Q column, GE Healthcare; Imamura et al., 1995). In addition to the further purification of each fraction, the screening of the DNA polymerase activity from the heat-stable protein library, made from *E. coli* cell extracts containing *P. furiosus* DNA fragments, revealed a new DNA polymerase gene (Uemori et al., 1997). The new DNA polymerase consisted of two proteins, the small and large subunits, and we named it DP1 and DP2. There two proteins are strictly required for both 5^′^–3^′^ polymerizing and 3^′^–5^′^ exonucleolytic activities *in vitro*. The genes encoding DP1 and DP2 are located in tandem on the *P. furiosus* genome and form an operon. Interestingly, this operon has a total of five genes, including a gene encoding a eukaryotic Cdc6/Orc1 protein (important for initiation of DNA replication) and a gene encoding a Rad51-like protein (involved in homologous recombination in Eukarya), in addition to DP1 and DP2 (**Figure 3**; Uemori et al., 1997). This was the first report of a eukaryotic-like initiator protein for DNA replication in Archaea. The amino acid sequences of DP1 and DP2 are not similar to those of any other DNA polymerases. After the discovery of this DNA polymerase, the total genome sequence of *Methanococcus jannaschii* was published as the first complete archaeal genome (Bult et al., 1996). One of the topics of this report was that only one DNA polymerase (family B) was found in the deduced amino acid sequences, in contrast to the three DNA polymerases, PolI, II, and III, in *E. coli* and several DNA polymerases in eukaryotic cells (Gray, 1996). We searched for homologous sequences of DP1 and DP2 in the *M. jannaschii* genome, and found them. The two genes were not present in tandem, but were located separately on the genome. We cloned and expressed them in *E. coli*, and demonstrated their polymerase and exonuclease activities *in vitro*. With this report, DP1 and DP2 became recognized as a novel archaeal DNA polymerase (Ishino et al., 1998). Three more total genome sequences were subsequently reported, and the genes for DP1 and DP2 were found in all them. Thus, this new DNA polymerase became more generally found in Archaea (Cann et al., 1998). Due to the lack of sequence homology to other DNA polymerases, we proposed a new family, family D, for this enzyme (Cann and Ishino, 1999).

**FIGURE 4:**
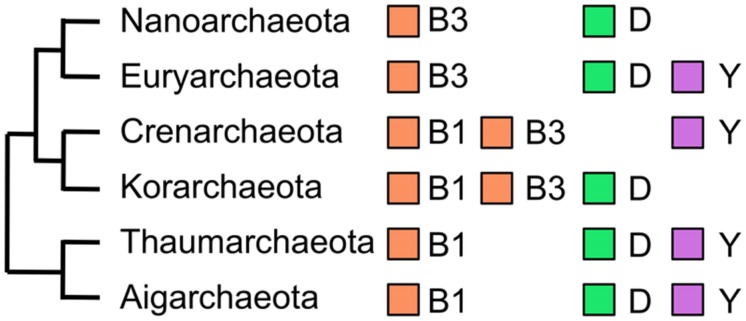
**DNA polymerases in Archaea.** The evolutionary relationships of six phyla in the domain Archaea are schematically shown with the DNA polymerases encoded in their genomes. The family B DNA polymerases from extrachromosomal elements were excluded.

In parallel to the identification of DNA polymerase activities in the cell extract of *P. furiosus*, we amplified a gene fragment for the family B DNA polymerase from the genomic DNA of *Pyrodictium occultum*, which grows at 105^∘^C, in an attempt to find a more heat-stable DNA polymerase than that from *P. furiosus*. By using a set of mixed primers based on the conserved sequences of motifs A and C in the family B DNA polymerase, a single band was amplified. However, two different fragments were found after the cloning and sequencing of the PCR product. The full-length sequences of both *pol*-like genes were cloned from the *P. occultum* genome by the primer walking method, and they were expressed in *E. coli*. Both of the gene products exhibited the heat stable DNA polymerase activity (Uemori et al., 1995). Unfortunately, the performance of these two enzymes in PCR was not better than Pfu polymerase, and we discontinued further research on them. However, this was the first report that an archaeal cell has two different family B DNA polymerases. It was an exciting discovery because three family B DNA polymerases, Polα, Polδ, and Polε, were known in eukarya, and so we proposed that plural family B enzymes were a common feature between Archaea and Eukarya. However, there is only one gene encoding a family B DNA polymerase in the *M. jannaschii* genome as described above. We subsequently found two family B DNA polymerases in *Aeropyrum pernix* (Cann et al., 1999), and thus the presence of two family B enzymes is not special for *Pyrodictium*, but is more general in Archaea. In the early stages of the total genome sequences, all sequences were from Euryarchaeota (*Archaeoglobus fulgidus, Methanothermobacter thermautotrophicus, Pyrococcus horikoshii*) and the determination of the genome sequence of a crenarchaeal organism was delayed until that of *A. pernix* was reported (Kawarabayasi et al., 1999). Taken together with the new knowledge at that time, it was predicted that euryarchaeal organisms have one DNA polymerase each from family B and family D, respectively, and crenarchaeal organisms have at least two family B enzymes in the cell. This overview of the distribution of DNA polymerases in Archaea is generally correct as shown in (**Figure 4**), which displays DNA polymerases in the archaeal phyla (subdomains) including newly proposed phyla from recent ecological research.

All of the original biochemical data for *P. furiosus* PolD from our group, including thermostability, strong primer extension and 3^′^–5^′^ exonuclease activity, showed that PolD is a suitable enzyme for PCR (Ishino and Ishino, 2001). However, PolD has not been commercially developed. Recent analysis of *Pyrococcus abyssi* PolD revealed that it is a suitable PCR enzyme (Killelea et al., 2014). On the contrary, PolD from *Thermococcus* sp 9^∘^N does not have any advantages as compared with the current commercially available PCR enzymes (Greenough et al., 2014).

## PROTEIN ENGINEERING OF THERMOSTABLE DNA POLYMERASES

Once PCR technology was established, efforts to improve PCR performance were pursued. At the early stage, hot start PCR was one of the big improvements for the specific amplification. An antibody against Taq polymerase was used to suppress its enzyme activity by specific antigen**–**antibody binding at the low temperature, and when PCR started from the denaturing temperature at more than 90^∘^C, the antibody became separated from the enzyme by heat denaturation. This hot start PCR method is generally effective to prevent non-specific amplification. For this purpose, another idea was tested. A chemical modification of Taq polymerase inactivated its enzymatic activity at low temperatures, but the modification can be released by high temperature resulting in activation of Taq polymerase to start PCR. This temperature-dependent reversible modification of the Taq protein led to the commercial product, AmpliTaq Gold, as the hot start PCR enzyme. Alternatively, a cold-sensitive Taq polymerase with markedly reduced activity at 37^∘^C, as compared with the wild type enzyme, was produced by site-directed mutagenesis, and this mutant is suitable for hot start PCR (Kermekchiev et al., 2003).

Taq polymerase is a family A enzyme, and is applicable to practical dideoxy sequencing. However, the output of the sequencing data was not ideal as compared with that from T7 DNA polymerase (known commercially as Sequenase; see below). An ingenious protein engineering strategy produced a mutant Taq polymerase that is more suitable for dideoxy sequencing than the wild type Taq polymerase. *E. coli* PolI and Taq polymerase discriminate deoxy- and dideoxynucleotide as substrates for the incorporation into the DNA strand, and therefore, an excess amount (50 to 1000-fold) of dideoxynucleotides must be present in the reaction mixture to stop DNA strand synthesis by their incorporation. For this property, the strength of each signal is not uniform, but is distinctly unbalanced. However, T7 DNA polymerase equally incorporates deoxynucleotides and dideoxynucleotides, and therefore, it is easy to adjust the reaction conditions to provide very clear signals (Tabor and Richardson, 1990). A mutant T7 DNA polymerase lacking the 3^′^–5^′^ exonuclease activity was developed as a commercial product, named Sequenase. A detailed comparison of *E. coli* Pol I and T7 polymerase revealed one amino acid that discriminates deoxy- and dideoxynucleotides, resulting in the successful conversions of the properties from PolI to T7 and T7 to PolI (Tabor and Richardson, 1995). This work was applied to Taq polymerase and a modified Taq with F667Y, which endows Taq with T7-type substrate recognition, was created (Tabor and Richardson, 1995). This enzyme was called Thermosequenase, and it became popular as the standard enzyme for the fluorescently labeled sequencing method (Reeve and Fuller, 1995).

Another target for the creation of a new enzyme by mutagenesis is an enzyme that is more resistant to PCR inhibitors in blood or soil, such as hemoglobin and humic acid. A mutant Taq DNA polymerase with enhanced resistance to various inhibitors, including whole blood, plasma, hemoglobin, lactoferrin, serum IgG, soil extracts, and humic acid, was successfully created by site-directed mutagenesis (Kermekchiev et al., 2009). The molecular breeding of *Thermus* DNA polymerases by using a direct evolution technique, compartmentalized self-replication (CSR; Ghadessy et al., 2001), also generated a PCR enzyme with striking resistance to a broad spectrum of inhibitors with highly divergent compositions, including humic acid, bone dust, coprolite, peat extract, clay-rich soil, cave sediment, and tar (Baar et al., 2011). Furthermore, enzymes with a broad substrate specificity spectrum, which are thus useful for the amplification of ancient DNA containing numerous lesions, were also obtained by the CSR technique (Ghadessy et al., 2004; d’Abbadie et al., 2007). Mutational studies of the O-helix of Taq DNA polymerase produced enzymes with reduced fidelity, which may be useful for error-prone PCR (Suzuki et al., 1997, 2000; Tosaka et al., 2001).

**FIGURE 5:**
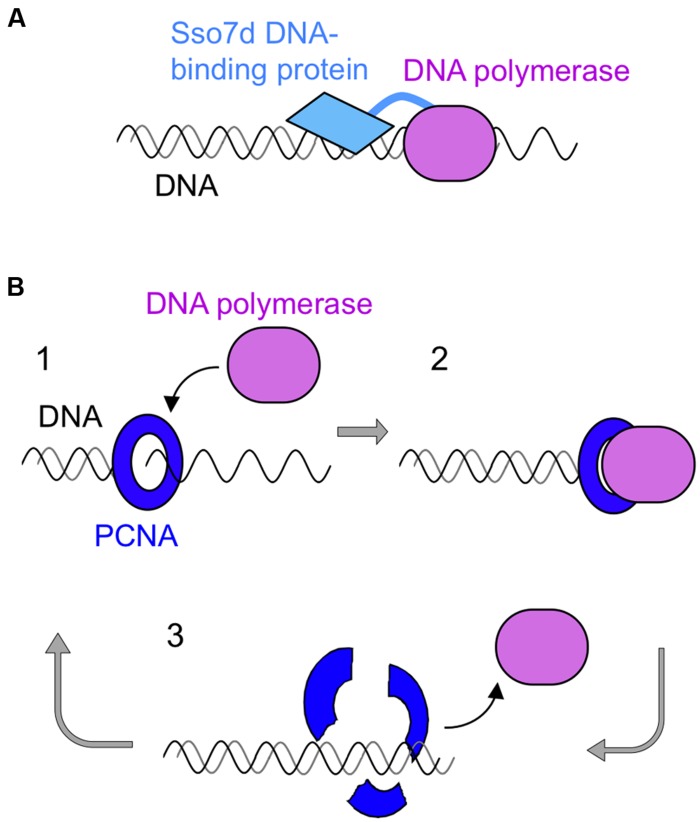
**Schematic diagrams of processive PCR using a family B DNA polymerase. (A)** The DNA binding protein, Sso7d, from *S. solfataricus* was fused with Pfu polymerase to confer high processivity to this enzyme.** (B)** PCNA-assisted PCR. 1, Self-loading of mutant PCNA on DNA; 2, PCNA-assisted processive DNA synthesis; 3, disassembly of the complex after DNA synthesis.

One successful strategy to produce improved DNA polymerases is the “domain tagging.” For example, new DNA polymerases were created by the flexible attachment of helix–hairpin–helix (HhH) domains of *Methanopyrus kandleri* topoisomerase V to the catalytic domains of Taq polymerase and Pfu polymerases. HhH is a widespread motif and generally functions on sequence-nonspecific DNA binding. These hybrid enzymes increased thermostability and became more resistant to salt and several inhibitors such as phenol, blood, and DNA intercalating dyes (Pavlov et al., 2002). This tagging strategy was also applied to φ29 DNA polymerase (de Vega et al., 2010) and *Bacillus stearothermophilus* DNA polymerase (Pavlov et al., 2012). Another successful example of the tagging strategy is the creation of commercial product “Phusion DNA polymerase” (**Figure 5**). This is a fusion protein of Pfu DNA polymerase and a DNA binding protein, Sso7d, from *S. solfataricus* (Wang et al., 2004). Sso7d has strong affinity to DNA, and it retains the fused Pfu DNA polymerase on the DNA once it starts DNA synthesis along with the template DNA strand. Phusion DNA polymerase compensates for the low extension rate of Pfu DNA polymerase while maintaining its high fidelity. This enzyme shows very high processivity and accurate PCR performance, and is now widely used.

Another idea to improve the processivity of the archaeal family B DNA polymerases was to use PCNA (proliferating cell nuclear antigen) as a processivity factor. The ring-shaped PCNA encircles the DNA strand and slides on it, and various binding proteins are attached to PCNA (Pan et al., 2011). DNA polymerase is a typical PCNA binding protein and it is connected to the DNA strand by PCNA during strand synthesis. This is why DNA polymerase shows highly processive DNA synthesis in the presence of PCNA. Based on this property of PCNA, scientists have searched for a thermostable PCNA for PCR with DNA polymerase. However, PCNA has not yet been successfully used for PCR. Unexpectedly, PCR is inhibited, rather than stimulated, in the presence of PCNA. We developed a PCNA-assisted PCR method, which is highly processive PCR with high fidelity, by using a mutant PCNA. Originally, we determined the crystal structure of *P. furiosus* PCNA (PfuPCNA; Matsumiya et al., 2001), and our continued research revealed that the intermolecular ion pairs between the protomers of PfuPCNA contributed to its ring stability (Matsumiya et al., 2003), which was greatly affected by the ionic strength of the solution. Mutations of the amino acid residues involved in the ion pairs clearly decreased its ring stability, but unexpectedly, a less stable mutant PfuPCNA enhanced the primer extension reaction of Pfu DNA polymerase *in vitro* (Matsumiya et al., 2003). Therefore, we applied the mutant PfuPCNA to PCR and successfully amplified DNA fragments up to 15 kbp with a markedly shorter reaction time, by Pfu DNA polymerase in the presence of a PfuPCNA mutant under conditions where Pfu DNA polymerase alone did not function (Ishino et al., 2012; Kawamura et al., 2012) This PCNA-assisted PCR (**Figure 5**) is also a successful example of processive PCR with high accuracy.

Because of the high sensitivity of PCR, very small amounts of carry-over contaminants from previous PCRs are considered to be one of the major sources of false positive results. The most common strategy to prevent carry-over contamination is to replace dTTP with dUTP during PCR amplification, thereby producing DNA containing uracil. Prior to initiating PCR, the PCR mixture is treated with Uracil-DNA glycosylase (UNG). During the initial denaturation step temperature is elevated to 95^∘^C, resulting in cleavage of apyrimidinic sites and fragmentation of carry-over DNA. One problem of the archaeal family B DNA polymerase to be used for this carry-over prevention is that they specifically interact with uracil and hypoxanthine, which stalls their progression on DNA template strands (Connolly, 2009). The crystal structure of the DNA polymerase revealed that read-ahead recognition occurs by an interaction with the deaminated bases in an N-terminal binding pocket that is specifically found in the archaeal family B DNA polymerases (Fogg et al., 2002). Due to this specific recognition of uracil, the archaeal family B DNA polymerases, including Pfu DNA polymerase and KOD DNA polymerase, are not suitable for carry-over prevention PCR. To conquer this defect, a point mutation (V98Q) was introduced into Pfu polymerase. This mutant enzyme is completely unable to recognize uracil, while its DNA polymerase activity is unaffected (Fogg et al., 2002; Firbank et al., 2008). Therefore, this mutant Pfu polymerase is useful for the carry-over prevention PCR. It is also useful for amplification of uracil-containing DNA, such as damaged DNA and bisulfite-converted DNA for epigenetic analysis.

## FUTURE PERSPECTIVES

Polymerase chain reaction initiated a revolution in molecular biology, and is now used daily not only in research, but also in the general human society. PCR is a complete technology, but more powerful and reliable enzymes for PCR are still desired. Notably, an enzyme with faster, longer, and more efficient extension ability, as compared to the properties of the current commercial products, will contribute to further improvements in PCR technology. In addition to these basic abilities, DNA polymerases that can incorporate various modified nucleotides, which are useful for highly sensitive labeling, are valuable for single molecule analysis. Mutations of the DNA polymerase itself, by site-specific or random mutagenesis, are effective ways to create modified enzymes with improved PCR performance or specific properties for *in vitro* DNA manipulations. An artificial evolution procedure also has attracted a great deal of attention, for the creation of DNA polymerases with novel activities (Brakmann, 2005; Henry and Romesberg, 2005; Holmberg et al., 2005; Ong et al., 2006). Our strategy of using environmental DNA as a genetic resource also works well to investigate the structure–function relationships of DNA polymerases. The region corresponding to the active center of the DNA polymerizing reaction, in the structural genes of Taq polymerase and Pfu polymerase, was substituted with PCR fragments amplified from DNAs within soil samples from various locations in Japan. The chimeric *pol* genes were constructed within the expression plasmids for the Taq and Pfu polymerases in *E. coli*. The chimeric enzymes thus produced, exhibited DNA polymerase activities with different properties (Matsukawa et al., 2009). The main focus for the future development of DNA polymerases is not on versatile enzymes, but rather on specialized enzymes suitable for individual purposes, including whole genome amplification, rapid detection of short DNA, new sequencing technologies, etc. Continued research on DNA polymerases may facilitate the invention of new genetic analysis technologies that are completely different from PCR or PCR-related techniques. The isothermal amplification without temperature cycling is more convenient and practical than PCR, and development of this type of technique has been actively performed (Gill and Ghaemi, 2008). Several methods practically utilized now are based on the strand displacement (SD) activity of the DNA polymerases. DNA polymerases from φ29 bacteriophage and *B. stearothermophilus* are the representative enzymes for the SD activity. A whole genome amplification using the SD activity of φ29 DNA polymerase is now especially useful for single cell analysis. Alternatively, helicase was applied for the dissociation of the double-stranded DNA from an idea to mimic DNA replication *in vivo* (Vincent et al., 2004). Although the helicase-dependent amplification (HDA) technique has not been practically used (Jeong et al., 2009), brushing up this technique may generate a powerful tool for genetic engineering.

## Conflict of Interest Statement

The authors declare that the research was conducted in the absence of any commercial or financial relationships that could be construed as a potential conflict of interest.
